# Retrogated spiral 3-directional myocardial phase velocity mapping in a single breath-hold

**DOI:** 10.1186/1532-429X-14-S1-W37

**Published:** 2012-02-01

**Authors:** Robin Simpson, Jennifer Keegan, Peter D Gatehouse, David N Firmin

**Affiliations:** 1 CMR Royal Brompton Hospital, Imperial College, London, London, UK

## Background

Myocardial phase velocity mapping studies have generally been acquired using Cartesian k-space coverage and respiratory gating [1,2]. Acquisition durations for high temporal resolution studies are therefore long and unpredictable and the use of navigators and prospective cardiac gating results in ‘dead-times’ in the cardiac cycle where imaging cannot be performed. We have developed a technique which combines highly efficient spiral k-space coverage with retrospective cardiac gating for 3D velocity mapping over the entire cardiac cycle within a breath-hold. The feasibility for rapid assessment of myocardial motion is demonstrated.

## Methods

The sequence (TR=30ms) consists of five interleaved spiral k-space paths (23ms). Reference and 3-directional velocity encoded data (15cm/s in-plane, 25cm/s through-plane) are acquired in consecutive cardiac cycles following a single dummy cycle (breath-hold duration 21 cardiac cycles). A black-blood suppression pulse (6ms) is output on alternate phases to reduce blood flow artefacts and retrospective gating allows full coverage of the cardiac cycle. Forty phases are reconstructed (reconstructed temporal resolution 19-31ms depending on heartrate) with an acquired spatial resolution of 2.4 x 2.4 x 8mm (reconstructed 1.2x1.2mm).Basal, mid and apical short axis slices were acquired in 6 healthy volunteers on a Siemens Skyra 3Tesla scanner. Radial, circumferential and longitudinal velocities were calculated pixel-wise and as an average over six semi-automatically segmented regions of the left ventricle.

## Results

**Figure 1:**
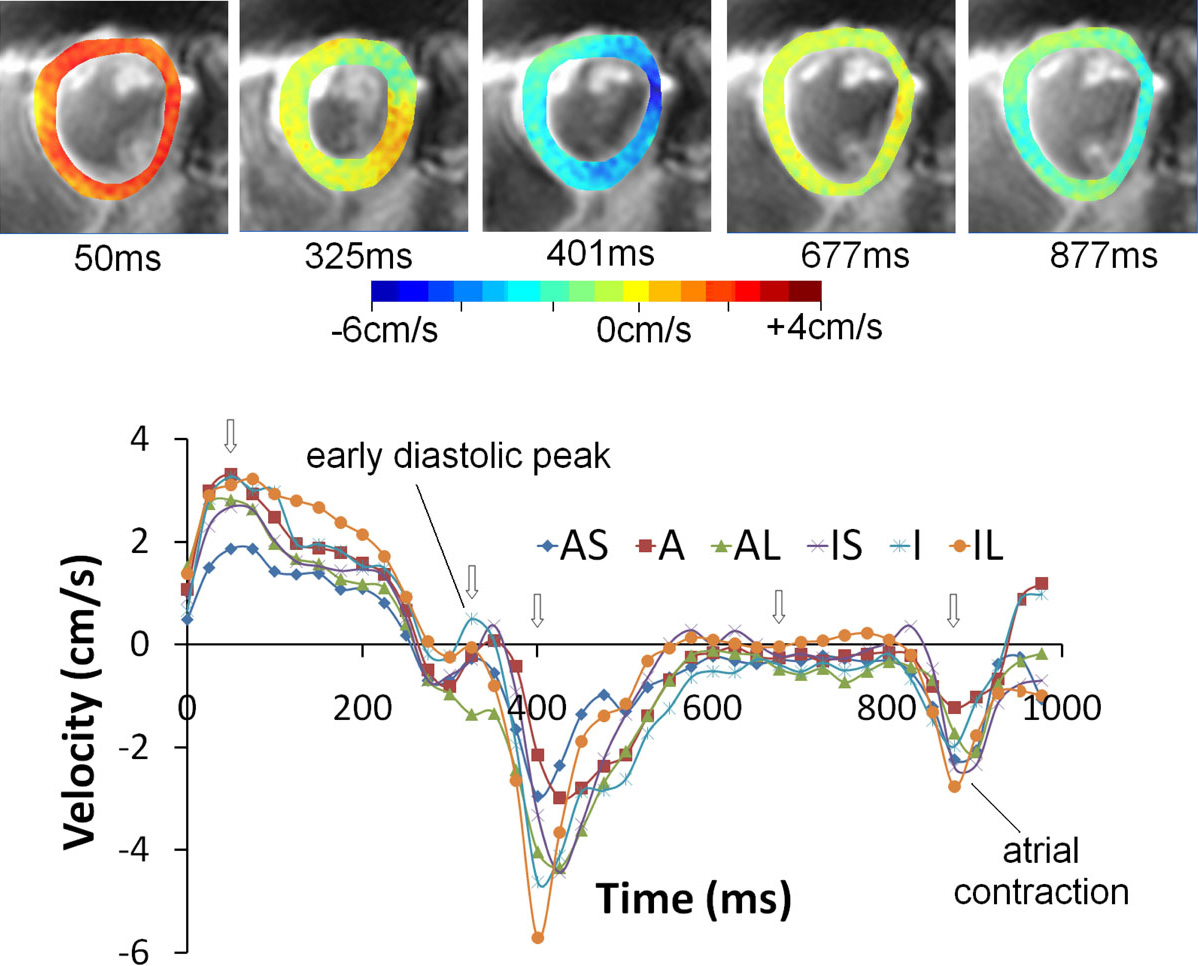
Magnitude images with colour-coded overlays representing radial velocities in the mid ventricular short axis slice of an example volunteer at five time points in the cardiac cycle (top) together with corresponding regional myocardial velocity time curves (bottom). The open arrows show the time points of the radial colour plots. Regional differences are most apparent in early-mid diastole. (AS = antero-septal, A = anterior, AL = antero-lateral, IS = infero-septal, I = inferior, IL = infero-lateral).

**Figure 2:**
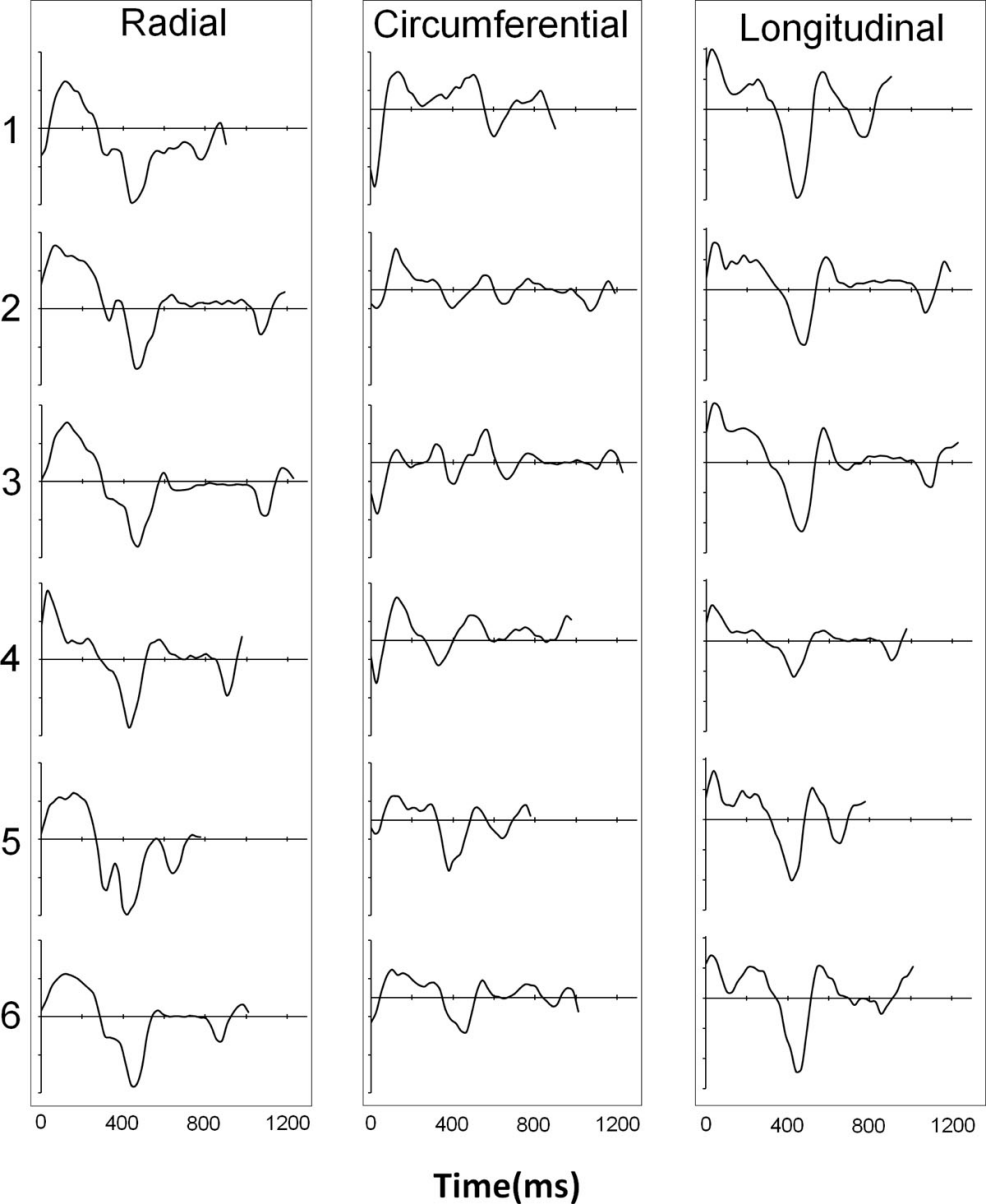
Global radial (left), circumferential (middle) and longitudinal (right) velocities over the entire cardiac cycle in the mid short axis slice in all 6 subjects. The longitudinal and radial temporal patterns show low inter-subject variability (apart from the length of diastole). Circumferential patterns are more subject-specific and also strongly dependent on the exact slice location [1]. (velocity scales: radial -4 to +4cm/s, circumferential -5cm/s to +3cm/s, longitudinal -9 to +6cm/s).

Figure 1 shows an example mid short axis dataset with colour-coded radial velocities at 5 time points in the cardiac cycle. The regional radial velocities are shown below and demonstrate the expected early diastolic biphasic pattern. The systolic time course is similar for all regions with regional variations becoming apparent in diastole. Ventricular expansion resulting from atrial contraction at the end of the cardiac cycle is clearly seen. Figure 2 shows global radial, circumferential and longitudinal velocities over the cardiac cycle for all six subjects. Inter-subject variability is low and the temporal patterns closely match previously published work acquired over much longer time intervals [1,2].

## Conclusions

We have developed a phase velocity mapping technique which allows 3D myocardial velocities to be acquired in a single breath-hold. The spiral k-space coverage results in reasonable temporal resolution (33ms) and the implementation of retrospective ECG-gating allows analysis of the entire cardiac cycle, including atrial contraction. Future work will include implementing an on-line correction map to reduce off-resonance blurring and parallel imaging to reduce the breath-hold duration.

## Funding

Heart Research UK grant:RG2584.
